# Bioengineering Stem Cell-Derived Glioblastoma Organoids: A Comprehensive Review

**DOI:** 10.3390/ph18121830

**Published:** 2025-12-01

**Authors:** Alexandra D. Avera, Yonghyun Kim

**Affiliations:** Department of Chemical and Biological Engineering, The University of Alabama, Tuscaloosa, AL 35487, USA; aavera@crimson.ua.edu

**Keywords:** organoids, bioengineering, stem cells

## Abstract

The development of novel therapeutics for deadly diseases such as glioblastoma (GBM) is bottlenecked by poor preclinical models. GBM is the most common and deadliest primary brain tumor in adults, with an average prognosis of 12–15 months, primarily due to its high cellular heterogeneity and treatment resistance from GBM stem cells. The advancement of in vitro models into organoids, three-dimensional tissue-like modeling systems, has been a promising approach to improving translational medicine for GBM. However, the critical tradeoff between technical convenience and physiological relevance threatens the integrity and reproducibility of GBM organoid (GBO) biomanufacturing. This comprehensive review breaks down and discusses the key features of GBM tumor microenvironment (TME), traces the advancement of in vitro models from two-dimensional cultures to three-dimensional stem cell-derived GBOs, evaluates the process through an engineering perspective (genetic, biochemical, biophysical, and process engineering), and addresses critical translational gaps. Reviewing trends over the last fifteen years in biomanufacturing approaches to GBOs revealed fundamental oversights that address previous review focuses on the limitations of organoids (i.e., maturity, vasculature, and immune defense). To summarize, GBO’s translational gap and reproducibility challenges are rooted in the prioritization of technical convenience over physiological relevance. To achieve clinical relevance, future GBO development must focus on transitioning to fully defined components (excluding animal-derived ECM), developing sufficiently large-sized constructs to recapitulate the full TME, and integrating non-destructive and enhanced functional readouts of the GBOs.

## 1. Introduction

Glioblastoma (GBM), formerly known as glioblastoma multiforme, is the most common and deadliest primary brain tumor in adults, with an average prognosis of 12–15 months [1,2]. GBM presents unique challenges, primarily due to its high cellular heterogeneity in the tumor microenvironment (TME) and due to resistance to treatment from GBM stem cells (GSCs) [3,4,5]. Organoids are tissue-like modeling systems that bridge the gap between the complex physiological relevance offered in animal tissue models and the availability and human translation of 2D culture systems [6]. The development of glioblastoma organoids (GBOs) has successfully recapitulated some spatial organization of the TME, including neurovasculature, necrotic core, and infiltrating rim, but they are often limited in their ability to functionally model these complex microstructures and others (i.e., excitatory neurons or immune system) that are essential for clinical translation [7,8,9,10,11]. The inability to advance GBO functionality is not rooted in technical impossibility, but in the field’s struggle with reproducibility and standardization.

This review critically evaluates the progression of GBO development through an engineering lens, aiming to define the necessary shifts required for the reproducibility needed for improved clinical relevance. Previous reviews on brain-derived organoids highlighted the limitations in modeling the complete microenvironment, including, but not limited to, neural activity, vasculature, and immune defense [12,13,14,15]. While these reviews revealed critical limitations in the biological translation of brain organoid modeling, the challenges in reproducibility and translation within a single lab and across other labs are often overlooked. Here, we summarize the parallels between healthy brain development and GBM development with the progression of in vitro modeling, then break down the current GBO generation methods from an engineering perspective. This summary was developed by selecting literature driven by thematic relevance over the last fifteen years and across three distinct fields: GBM/brain biology, organoid engineering, and biomanufacturing. A broad review of biomanufacturing approaches of organoids allows for insights into where variability can occur. This review discusses the preference for technical convenience, leading to fundamental oversights that compromise reproducibility and physiological relevance. Specifically, these include avoiding sufficiently large length scales required to model the true complexity of the neurovasculature and necessary distance leading to necrosis, and the overreliance of undefined components—namely extracellular matrix (ECM) hydrogels—that fundamentally obscure the study of critical cell-ECM dynamics. Ultimately, this review reveals clear engineering parameters required for GBO standardization needed for translational reproducibility.

## 2. The Development of Glioblastoma Tumor Microenvironment Parallels in Brain Healthy Brain Neurogenesis

GBM is a complex brain tumor that can rapidly occur with a sudden onset or develop from a lower-grade glioma (LGG), leading to a typical two-year survival rate of 27–30% despite the best available standard of care [1,16,17,18,19,20,21]. This poor prognosis is often attributed to the lack of a deep understanding of the tumor microenvironment (TME), especially during the development from the glioblastoma stem cell (GSC) into a fully formed tumor tissue. GSCs are mutated glial cells, non-neuronal cells that protect neurons in the central nervous system [22,23]. The GBM TME is composed of microstructures such as vasculature, necrotic core, and infiltrating rim that make up the whole tumor system [4,5,24,25]. Each of these microstructures is composed of different cell types with varying morphological features, expression patterns, and even secreted extracellular matrix (ECM). The ECM is an intricate network of biopolymers (e.g., collagen, fibronectin, and hyaluronic acid) that surrounds cells, guiding their communication, migration, and fate [26,27]. Developing a physiologically relevant model that incorporates all of these variables has been a leading challenge in GBM treatment progression, but the complexity of the development of the GBM TME shares striking similarities with healthy neurogenesis, and the rise of neurons and glial cells in human development.

### 2.1. Healthy Neurogenesis

Healthy neurogenesis has previously been reviewed in the cited references [28,29,30]. In short, neurogenesis begins in the embryo when totipotent (embryonic) stem cells self-renew rapidly, form a blastocyst structure, and differentiate into pluripotent stem cells (PSCs) that organize into the three germ layers: endoderm, ectoderm, and mesoderm. The ectoderm gives rise to the neuroectoderm after the PSCs differentiate into neural stem cells and neural progenitor cells, often recognized as radial glial cells (RGs). The parallels between GBM and healthy brain development begin here, where both processes rely on stem cells to differentiate into various cell types and are guided by an intricately organized ECM [31]. The primary difference is that healthy neurogenesis is a tightly regulated, ordered, and spatiotemporally controlled process that contributes to brain function [32]. RGs are highly polarized and seek to provide brain function by giving rise to intermediate progenitor cells, apical RGs, and basal RGs which later differentiate into mature neurons, astrocytes, or oligodendrocytes [29]. GSCs seemingly seek to generate an entirely new brain tissue by sustaining uncontrolled growth and invasion of healthy tissue and diverting necessary nutrients to the tumor through vascular formation [33,34,35]. Furthermore, one misunderstood and often overlooked microstructure of the GBM TME in in vitro modeling systems is the ECM and its role in GSC development into tumor tissues [33].

### 2.2. Extracellular Matrix (ECM)

Emerging studies indicate that ECM is not merely an inert scaffold; rather, it is a dynamic and interactive tissue component that guides cell structure, function, and fate [36,37,38]. In healthy neurogenesis of embryonic brains, tenascin-C (TNC), hyaluronic acid (HA), and neurocans are highly expressed, allowing for easy cell proliferation, axonal growth, and neuronal migration due to their anti-adhesive qualities [39,40,41]. In contrast, healthy adult brains are highly expressive of tenascin-R and brevican to support neural excitation instead [42,43,44,45]. GBM ECM expression patterns closely mimic those of healthy embryonic brain tissue, suggesting that GSCs and their differentiated tumor cells are redesigning the local ECM to infiltrate healthy tissue and expand GBM tissue [5,37,46]. Other ECM subtypes are present in both healthy and GBM tissue according to the microenvironment structure. For example, vascular structures are supported by the basement membrane highly composed of laminin, fibronectin, collagen IV, and perlecan [47,48,49]. Perineuronal nets (PNNs) contain chondroitin sulfate proteoglycan (CSPG) ECMs that support mostly interneurons [50,51]. Studies indicate ECMs related to the basement membrane are generally upregulated, while PNNs are generally downregulated when comparing GBM tissue to healthy brain tissue [48,52,53,54,55]. Notably, the ECMs that are most upregulated in GBM are also associated with increased stiffness, likely due to the large fibrous glycoprotein composition (i.e., collagen IV) or high crosslinking capabilities (i.e., HA) [55,56,57,58,59,60,61,62,63,64]. The extensive remodeling of ECM highlights a significant challenge for researchers seeking to develop physiologically relevant modeling systems of GBM and its TME in vitro [65]. Beyond the ECM, the neurovascular unit (NVU) is another critical microstructure that is often misunderstood when developing GBM modeling systems.

### 2.3. The Neurovascular Unit (NVU)

The neurovascular unit (NVU) is a systemic network of blood vessels responsible for delivering essential nutrients to the central nervous system (CNS) and is well-described in previous reviews [66,67]. The NVU is composed of arterioles (smallest arteries that deliver oxygenated blood) and venules (smallest veins that export deoxygenated blood) that are interconnected with capillary beds (weblike structures of the smallest and simplest blood vessels, capillaries, to exchange gases and nutrients throughout the brain) [65,68,69]. Each of these blood vessel types (i.e., arteriole, venule, capillary) are lined with endothelial cells (ECs) that are supported by pericyte cells (PCs) and astrocyte cells (ACs) that communicate between the blood vessels and neurons [53,70,71,72]. This EC-PC-AC structure is referred to as the blood–brain–barrier (BBB), which acts as a dynamic interface for tight nutrient diffusion regulation while also protecting the CNS from toxins and pathogens [47,53,73,74,75,76]. Since GBM is often derived from a mutated AC turned GSC, the NVU is a critical microstructure to model to understand the transformation of the GSC into a GBM tissue. GSCs are capable of transdifferentiating into ECs causing uncontrolled vascularization and sprouting angiogenesis [72,77,78]. The uncontrolled formation of blood vessels is often rapid, leading to “leaky” blood vessels determined to infiltrate healthy blood vessels and divert essential nutrients to the tumor [68,77,79,80,81]. Common in vitro studies of the BBB mimic that of the capillary bed due to the capillary’s simple monolayer EC structure and small inner diameter (4–10 μm) [82,83]. However, the crosstalk between GBM cells and the entire NVU is often credited with the tumor’s invasive and aggressive nature. Therefore, it is imperative that in vitro modeling systems work to include the NVU.

### 2.4. The Brain’s Immune System

The brain’s innate immune system, primarily composed of microglia, plays a complex dualistic role in both healthy brain function and GBM progression [84]. In the healthy CNS, microglia are key sentinels that maintain tissue homeostasis, prune synapses, and clear cellular debris [52,85,86]. However, in the presence of a GBM tumor, these cells undergo a phenotypic shift, becoming tumor associated microglia (TAMs) that are differentiated by the cancer cells [85]. TAMs are a dominant non-malignant cell population within the GBM tumor and can secrete factors that promote tumor cell proliferation, survival, and immunosuppression [85]. Additionally, other immune cells from the periphery, such as macrophages, are recruited to the tumor site and similarly contribute to the pro-tumorigenic environment.

The intricate crosstalk between GBM cells and these immune components begins with the tumor cells themselves, as genetic alteration in GBM cells, notably NF1 deficiency, can drive the recruitment and infiltration of microglia macrophages [86]. GBM cells further establish the immunosuppressive microenvironment by secreting factors like CLL2 to recruit CCR2+ myeloid cells, and CSF1 to promote myeloid cell differentiation and survival [85,87,88,89,90]. In response to immune pressure, GSCs can undergo epigenetic immunoediting, acquiring myeloid-affiliated transcriptional programs that facilitate immune evasion by reshaping the immune TME [85,88]. Reciprocally, macrophages actively drive the phenotypic plasticity of GBM cells, inducing their transition into a mesenchymal-like state [86,91]. This transition is often mediated by macrophage-derived oncostatin M (OSM), which binds to receptors on GBM cells and activates STAT3 signaling [91]. This interplay results in a potent immunosuppressive niche, where TAMs and microglia contribute to immunosuppression by producing high levels of arginase, depleting arginine necessary for T-cell function [87,88]. This complex interplay is a major drive of therapeutic resistance, as the immune cells create a protective barrier around the tumor [86,87,88,89]. Therefore, any in vitro model that aims to accurately recapitulate the GBM TME for drug testing or mechanistic studies must include a functional immune component.

## 3. Critical Limitations in Physiological Relevance Have Triggered the Advancement of In Vitro Modeling Systems of Glioblastoma

Prior to the 1990s, researchers followed the stochastic model of cancer, which theorized that every cell within a tumor was capable of unlimited proliferation and could initiate a new tumor randomly, triggered by genetic mutations [16,92]. The goal of cancer treatment then was to simply kill as many cells as possible via radiation or chemotherapy. While this aggressive approach reduced the bulk of the tumor cell population, patient outcomes were still low due to tumor recurrence. In 1997, researchers discovered clear evidence of a residual cell population responsible for tumor recurrence and the existence of a cellular hierarchy in acute myeloid leukemia [93]. These residual therapy-resistant cells were later referred to as cancer stem cells (CSCs) [94,95]. The CSC theory postulates that cancer can only be sustained by mutated stem cells that exhibit uncontrolled self-renewal or differentiation into tumor-supporting cells. This theory quickly gained traction in the field of GBM in the early 2000s, leading to the establishment of GSC cell lines [23,92,96,97,98]. The therapeutic approach to GBM has since changed to target the GSC population.

### 3.1. Two-Dimensional (2D) In Vitro Systems

Early glioblastoma research was largely conducted using two-dimensional (2D) monolayer cell cultures of GSCs [99]. This conventional method involves growing a single layer of cells on a rigid, flat plastic surface, often made of polystyrene. Tissue culture polystyrene (TCPS) flasks offer the advantages of being cost-effective, easily reproducible, and simple to maintain [100]. Researchers have used this method with established GBM cell lines, such as U87 and U251, as well as with patient-derived GSCs [17,101,102]. While these models provided initial insights into simple GSC interactions that affect cellular proliferation and viability, it had little effect on patient outcomes. GSC-targeted therapies were developed but were seemingly not being delivered to GSCs in vivo [23]. As the CSC theory was further expanded, researchers discovered that CSCs and GSCs alike have a preferred niche where they reside [103,104]. The GSC niche is part of the TME, typically within the NVU, away from the infiltrating rim and dying necrotic core, but within nutrient diffusion limitations (100–200 μm) from the blood vessels [4,105]. The discovery of this preferred niche highlighted the critical limitations of 2D monocultures in recapitulating the complex in vivo TME. Cells grown in a monolayer lose their natural three-dimensional (3D) morphology, polarity, and key intercellular signaling pathways that are crucial for tumor behavior [25,106]. The flat surface also fails to mimic the mechanical properties of the brain’s ECM, a key regulator of GBM cell migration and aggressiveness. Furthermore, 2D cultures are often monocultures, meaning that they lack the diverse cellular components of the TME, such as the NVU and the immune system that are protecting the GSCs from these targeted therapies [74,106,107,108,109]. Many solutions have been offered to overcome these limitations such as surface treating the TCPS with a hydrogel to expose the cells to a softer, ECM-like environment [110,111,112,113,114]. Guided GSC differentiation into other cell types was attempted either biochemically (i.e., fetal bovine serum), biomechanically (i.e., adjusting TCPS stiffness or exposing cells to fluid shear stress), or genetically. However, there remains a significant gap between promising clinical findings and poor clinical outcomes, driving the need for more physiologically relevant modeling systems.

### 3.2. Three-Dimensional (3D) In Vitro Systems

Following the limitations of 2D models, researchers began to explore 3D cell culture systems, with spheroids emerging as one of the most prominent approaches. Spheroids are self-assembled, non-adherent, spherical clusters of cells that mimic the in vivo cellular arrangement and cell-to-cell interactions of solid tumors more closely than a monolayer [54,115,116,117]. This model gained traction as it was an improvement from 2D cultures to provide a more physiologically relevant system for studying GBM. Spheroid culture can be achieved using various methods, including low-adhesion plates, hanging drop techniques, or scaffold-based systems [118,119,120]. These methods promote cell aggregation, allowing GSCs to maintain their native morphology, polarity, and expression of stemness markers, all of which are lost in 2D culture [121,122]. Unlike the monoculture approach of 2D, spheroids can also incorporate other cell types from the TME, such as ECs or fibroblasts, enabling the study of critical cell–cell interactions [38,73]. Moreover, the 3D structure of spheroids creates gradients in oxygen, nutrients, and waste products, which are more representative of the in vivo TME [123,124,125,126]. Spheroids also display differential gene expression and enhanced resistance to a chemotherapy and radiation, better reflecting the therapeutic resistance seen in patients. While spheroids represent a significant advancement over 2D models, they still fall short in fully recapitulating the complexity of the GBM TME. They lack the full complement of cellular diversity found in the brain, such as immune cells and neurons, and do not capture the intricate biophysical cues and spatial organization of the brain’s ECM [127,128,129].

### 3.3. Organoids

**Figure 1:**
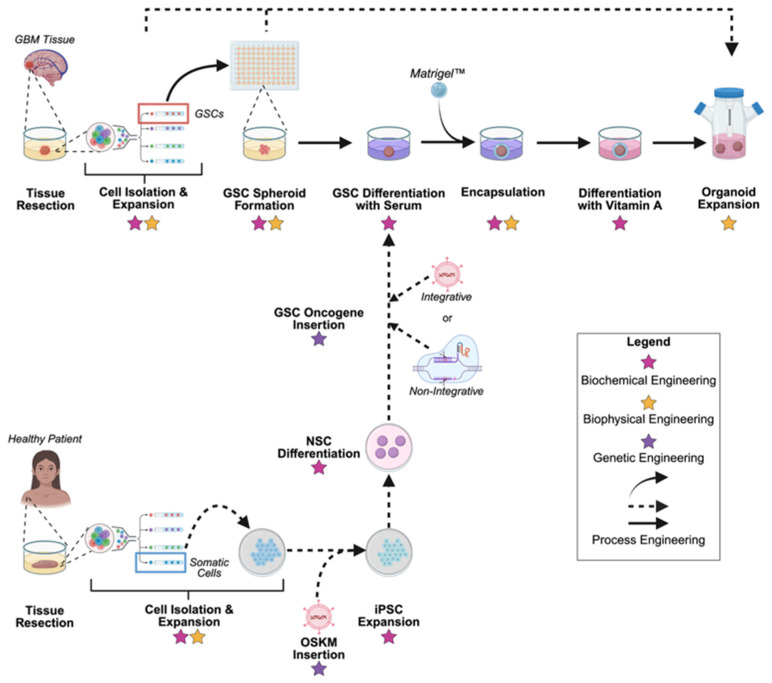
Current Trends in Biomanufacturing of Glioblastoma Organoids (GBOs). Current methods for producing GBOs require a combination of genetic, biochemical, biophysical, and process engineering. The most popular biomanufacturing process for GBOs is at top. Dashed arrows indicate other “bypass” processes for biomanufacturing GBOs, while solid arrows indicate the preferred process. The bypass process from GSCs directly inoculated into a bioreactor and spheroids directly inoculated into a bioreactor for organoid expansion are in the following references, respectively [8,46]. The bypass process involving healthy patient tissue can be found in the following references [138,139]. All steps prior to the organoid expansion step are grown in static conditions in tissue culture polystyrene with stiffnesses of >10^9^ Pa and largely overlook the biophysical influence. Future biomanufacturing of GBOs should consider the effects of biophysical engineering earlier in this process. GBOs developed from these processes are still largely limited in functional neurons, the neurovascular unit, and immune system modeling. Created in BioRender. Avera, A. (2025) https://BioRender.com/fv7sm9n.

The advancements of spheroid models and the CSC theory have led to a new 3D modeling system called organoids. Glioblastoma organoids (GBOs) are 3D tissue-like modeling systems that can be tissue-derived or GSC-derived. It encapsulates many of the cell and ECM subtypes found within the GBM tissue. Thus, organoids offer to bridge the gap of physiological relevance between cell culture and tissue culture systems, while still maintaining the relative accessibility of cell cultures [130,131]. Tissue-derived organoids are limited in accessibility compared to GSC-derived organoids as they require large freshly excised patient tissue [132]. Thus, this review will focus on GSC-derived organoids from here on (Figure 1). The most popular biomanufacturing process for GBOs closely mimics that of cerebral organoids [133,134,135]. To summarize this process: GSCs are sourced, formed into spheroids, differentiated in fetal bovine serum (FBS)-containing media, encapsulated in an exogenous ECM hydrogel (i.e., Matrigel™), then cultured in a different differentiation media before inoculating the GBOs in a bioreactor under sublethal shear stress to enhance nutrient diffusion [7,135,136]. Moreover, organoids can be co-cultured with other cell types, such as healthy CNS cells, or other tissue-like systems, such as gut organoids, to study the brain’s complex cellular interactions both within and outside the CNS [10,11]. While organoids are a powerful tool for translational research and personalized medicine, they face looming challenges because the biomanufacturing process of GBOs is highly laborious and not standardized, leading to reproducibility issues [12,137]. Lastly, GBOs are often criticized for their inability to model the fully matured GBM tissue with functionalized TME components. So far, it has proven difficult to advance the development of GBOs without first solving the issue of reproducibility.

## 4. Bioengineering Perspectives on Glioblastoma Organoid Fabrication and Control

The U.S. Food and Drug Administration (FDA) announced a plan to phase out animal testing requirements for monoclonal antibodies and other drugs after years of supporting the 3R principle (replace, reduce, and refine) [140]. In the place of animal testing, the FDA seeks to utilize advanced computing simulations and human-based lab models, specifically calling our lab-grown organoids [141,142]. Now, more than ever, is the time for the adoption of GBOs in drug development processes, but the adoption is slow and tedious. A key barrier preventing the widespread adoption of GBOs is the lack of reproducibility of biomanufacturing protocols. Addressing this challenge reveals the overwhelming variables involved in GBO production, ranging from the earliest stages of GSC sourcing to the final culture environment. Here, we classify the varying perspectives bioengineers must consider in the production and scale-up process of GBOs from stem cells.

Generating stem cell-derived GBOs begins with sourcing the GSCs. Recalling that GSCs are mutated glial cells, isocitrate dehydrogenase (IDH) enzyme wildtype mutation accounts for approximately 90% of primary GBM [101,143]. GSCs are frequently identified with epidermal growth factor receptor vIII (EGFRvIII) truncation amplification or mutation, phosphatase and tensin homolog (PTEN) deletion or mutation, and telomerase reverse transcriptase (TERT) promoter mutation [23,34]. The other 10% of GBM is identified as an IDH-mutant GBM, typically arising from LGGs, with genetic mutations in isocitrate dehydrogenase 1 (IDH1), tumor protein P53 (TP53), and alpha-thalassemia/intellectual disability X-linked (ATRX) [19,30]. Other important GSC identifiers are increased expression in SOX2, GFAP, and CD133 [144,145,146,147]. Patient-derived GBOs offer a personalized approach to modeling this deadly disease. Thus, patient-derived GSCs are sourced directly from a patient during initial biopsies or surgical resection of the tumor. In short, tumor tissue is resected, immediately placed in sterile transport media, washed, and minced for enzymatic digestion to break down its ECM, then mechanically dissociated and filtered with a cell strainer to ensure low-density seeding for neurosphere/serum-free culture expansion [148]. Eventually, the neurospheres are dissociated into single cells and sorted to isolate the mutations found in GSCs. There are many iterations of this general process with varying levels of reported reproducibility (30–96%) across different patients [148]. The variations in GSC sourcing directly from patients can be viewed through the perspectives of biochemical engineering and process engineering, both of which will be discussed in a later section.

### 4.1. Genetic Engineering

A crucial feature of GBOs is their easy accessibility compared to tissue models, but sourcing patient-derived GSCs from the tumor tissue is a challenging feat. Therefore, many researchers have turned to genetic engineering as an alternative method for sourcing and developing GBOs. Genetically engineered GSCs rely on healthy embryonic stem cells (ESCs) or induced pluripotent stem cells (iPSCs) as a starting material [122]. In general, these stem cells are first differentiated into NSCs or NPCs (the proposed cell of origin for GSCs). Utilizing non-integrative approaches (i.e., CRISPR-Cas9) or integrative approaches (i.e., lentiviral or retroviral vectors), key oncogenic drivers like EGFRvIII or PTEN deletion are introduced [11,34,149,150]. The overwhelming preference leans towards iPSC-derived GSCs due to patient-specificity and to avoid the regulatory and ethical hurdles faced with using ESCs. However, it would be naïve to ignore the epigenetic memory (ability to retain original cell characteristics) iPSCs maintain during reprogramming and following directed differentiation that ESCs do not face [149,151,152,153,154]. There are many variables to consider in the process of generating GSCs from iPSCs. First, somatic cells such as fibroblasts are sourced and reprogrammed by inserting and inducing overexpression of the four stem cell factors: OCT3/4, SOX2, KLF4, and MYC (OSKM) [155]. Upon successful insertion of OSKM, the cells are expanded on feeder cells (i.e., mouse embryonic stem cells) or exogenous ECM-coated plates with defined pluripotent media. Directed neural differentiation is initiated to the iPSCs through various media formulation steps, typically including serum. Finally, the cells undergo the genetic mutations specific to GBM (PTEN deletion/knockout, EGFRvIII expression, TERT promoter mutations) and are grown in serum-free NSC/GSC formulated media [54,153].

The challenge of generating GSCs from iPSCs primarily centers on balancing efficiency with genetic fidelity. The discovery of iPSCs by Yamanaka and colleagues in 2007 initially relied on integrative genetic engineering via viral vectors, which offered 50–95% efficiency in establishing iPSC lines [155]. However, integrative genetic engineering approaches carries the risk of random, non-specific mutations due to the integration of viral DNA into the host genome, which is highly undesirable when modeling the already unpredictable nature of GBM [156,157]. Notably, the organoid field is remarkably open and quick to adopt emerging technology. Shortly after the initial publication and popularization of cerebral organoids utilizing ESCs and viral vector-derived iPSCs in 2013, many researchers began adopting non-integrating genetic engineering methods such as CRISPR-Cas9 [11,135,150]. While CRISPR-Cas9 offers noticeably higher precision in gene editing with a reduction in random side effects, its typical efficiency is 5–30% [154,158]. The trending adoption of CRISPR-Cas9 for introducing oncogenic drivers, despite the lower yield, indicates a clear preference for predictability and genetic control over efficiency in the creation of rigorous, defined GBOs. Ultimately, for these genetically defined GSCs to successfully differentiate and form a viable GBO structure, the focus must shift from the genomic and transcriptomic instruction to the culture environment, necessitating a closer analysis of biochemical engineering perspectives.

### 4.2. Biochemical Engineering

The biochemical environment of GBOs can be broken into the composition of the culture medium and the ECM scaffolding used for 3D growth. Thus, biochemical engineering perspectives must be applied as early as sourcing tissue for GSC sourcing. The foundational challenge for biochemical engineers is moving away from undefined animal-derived products towards defined, chemically products [159]. In the case of cell culture media, a critical balance must be maintained: it must contain growth factors (i.e., EGF, and FGF) necessary to maintain the GSC population while simultaneously enabling the differentiation of other cells (i.e., neurons, glial, and endothelial cells) that make up the TME. Furthermore, ECM hydrogels are a major source of variability [159]. The choice of hydrogel scaffolds dictates the final GBO morphology, cellular fate, and structural organization. Matrigel™ is commonly used as an ECM hydrogel because it is mouse sarcoma-derived and laminin rich [160]. However, it is poorly defined with notorious batch-to-batch variation [160,161]. Moreover, the ECM of GBM is highly heterogeneous and rich in hyaluronic acid, collagen IV, fibronectin, and tenascin C. Fully defined and tunable synthetic, peptide-based, or modular biomaterials may substitute Matrigel™, but often have less influence on cells. Matrigel™, or similar tissue-derived hydrogels, are often still preferred by cells, and subsequently by scientists. The cellular preference is likely due to the epigenetic memory of the cells, preferring the naturally signaling molecules (i.e., growth factors) and the heterogeneity of tissue-derived ECM [161,162]. Defining ECM hydrogels and cell culture media must also be considered from the biophysical engineering perspective.

### 4.3. Biophysical Engineering

Biophysical engineering is essential for replicating the mechanical and transport conditions of in vivo GBM tissues, and it can also be broken down into two general categories: tissue stiffness and fluid shear stress of culture conditions. Cells exhibit a mechanical memory, similar to epigenetic memory, where prior exposure to physical cues such as matrix stiffness and fluid shear stress dictates the cell’s fate [110,163]. Healthy brain tissue is amongst the softest in the human body, with a reported compressive modulus of 0.1–2.0 kPa [164,165,166]. GSCs are known to retain their stemness and promote invasion in these softer environments. Conversely, the overall stiffness of a resected GBM tumor is reported to be 0.5–16 kPa [125,167,168,169]. The duality of the stiffness—a soft GSC niche and tumor core versus a stiff infiltrating rim—makes it challenging to recreate in vitro, necessitating precise microscale control over matrix tunability [167,170]. The introduction of 3D bioprinting has partially addressed these concerns but is often limited to 100 μm features, as opposed to the 0.1–1 μm resolution required for truly mimicking the native environment [171,172]. Moreover, the GSC niche is known to be tucked between blood vessels within nutrient diffusion limitations [103,172,173,174]. The location of the GSC niche suggests that GSCs are typically exposed to some fluid shear stress from the interstitial flow generated by blood vessels, estimated to be in the low physiological range of 0.01 to 0.1 Pa [175]. The introduction of shear stress in culture is mimicked via bioreactors, but the wide variety of bioreactors leads to varying and often non-uniform distributions of shear stress induced on the cells (Table 1). Thus, to overcome the size and uniformity constraints imposed by these biophysical limits, the focus must move to large-scale technological solutions rooted in process engineering.

### 4.4. Process Engineering

Addressing the constraints imposed by genetic, biochemical, and biophysical requirements necessitates the application of process engineering. This discipline serves as the crucial bridge, ensuring that specifications established early in the process (such as GSC sourcing) do not negatively interfere with later steps (such as GBO structural assembly). Process engineers are tasked with resolving operational variability through automation or high-throughput cell culture to standardize and screen every logistic parameter [186]. These parameters include cell culture geometry, shear stress, mechanical stiffness, the precise timing, pH, and temperature during enzymatic digestion, and strict control over the supply chain, defining the required lot, purity, and concentration for all chemical components [187,188,189]. Furthermore, automation is key to defining and maintaining ideal bioreactor specifications—the size, working volume, and mechanism of shear (rolling, shaking, or spinning)—to ensure uniform mass transport without inducing mechanical damage to the media supplements. While ideal for scale, the stirred-tank bioreactor’s variation in distribution of shear stress inherently compromises organoid reproducibility. To guarantee fidelity and consistency, process engineers implement real-time quality control (QC) checks, which are critical steps for verifying that key parameters are within tolerance. Ultimately, the integration of these automated QC steps couples with closed-loop feedback systems that continuously monitor and adjust critical process variables, representing the highest level of standardization and bridging directly into the domain of systems engineering.

## 5. Translational Gaps in Glioblastoma Organoid Development

After re-evaluation of current GBO biomanufacturing approaches, this section highlights three non-biological failure points that would invalidate even a biologically perfect model (i.e., future organoid models with extra technological approaches). Existing reviews have primarily focused on the biological translational gaps (i.e., failure to replicate treatment response or immunological interactions) and suggested the integration of new technologies to overcome these barriers. The final, crucial phase in GBO development shifts the focus from sophisticated development to pragmatic translation, revealing three often overlooked hurdles that must be overcome for clinical and commercial adoption: standardization, enhanced readouts, and logistics. Each of these elements determines the model’s reproducibility and accessibility. First, standardization is necessary to ensure data comparability. Without unified standard operating procedures (SOPs) that define everything from patient tissue dissociation to final ECM content, a GBO produced in one facility cannot be confidently compared to one produced in another. This lack of protocol fidelity severely limits large-scale drug screening efforts. Second, the model’s clinical value hinges on enhanced readouts. It is not enough for the organoid to merely be viable; assays must be established that quantitatively measure the key functional pathologies of the disease, such as GSC invasion distance, the specific mechanisms of drug penetration into the core, and the rate of resistance development. These metrics are required to provide truly predictive clinical data. Finally, logistics must be solved for global utility. Since GBOs are live, metabolically active tissues, challenges surrounding cryopreservation and reliable shipping must be addressed to ensure that the model’s structural and functional integrity is maintained throughout transit to distant pharmaceutical partners to treatment centers. Solving these three translational gaps are the final prerequisite for establishing GBOs as a robust, industrial biomanufacturing product.

### 5.1. Standardization

The primary prerequisite for moving glioblastoma organoids (GBOs) into clinical and industrial settings is comprehensive standardization, a challenge the NIH is actively addressing by establishing the nation’s first dedicated organoid development center called the Standardized Organoid Modeling (SOM) Center (announced on 25 September 2025) [190,191]. While the implementation of SOPs will resolve many of the basic reproducibility challenges, true standardization requires the establishment of quantifiable QC metrics. For GBOs, these metrics extend beyond basic viability to include relative transcriptomic and proteomic expression patterns. The NIH plan seeks to resolve the current crisis of reproducibility by having these centers perform rigorous benchmarking by testing and validating different published protocols against regulatory standards set by the FDA, Organization for Economic Co-operation and Development and Interagency Coordinating Committee on the Validation of Alternative Methods [190,191].

To establish a “standard GBO”, the process must define key operational criteria. First, the use of well-characterized GSC lines is critical for minimizing input variability. Furthermore, the GBO developmental trajectory may benefit by closely mimicking human neurogenesis patterns as previously published [28,29,30]. This involves defining developmental milestones (e.g., specific GBO size and clear patterns of cell groupings with ECM expression) at timed intervals, which will initially be quantified using destructive multiomics (e.g., transcriptomics, lipidomics, and proteomics) and histochemistry. To track clone lineage and ensure batch-to-batch consistency, genetic barcoding provides a quantitative method for monitoring clonal selection throughout the manufacturing process. As technology in enhanced readouts progresses (Section 5.2), standardization of GBOs will become a much easier task to tackle. The integrity of the entire translational pipeline is thus dependent on the rigor with which the field selects and validates appropriate regulatory standards for all organoid and subsequent GBO models.

### 5.2. Enhanced Readouts

While quantitative QC measures solve the reproducibility issue, they add a significant strain to accessibility because many of the most informative assays are inherently destructive. This limitation makes it necessary to prioritize and develop enhanced readout approaches for organoids. For instance, non-invasive measurements of cell number, %-viability, and wet/dry mass can be indirectly quantified by establishing a standard growth curve for GBOs, often defined through the projected area from routine imaging and normalizing to destructive weight and viability data. More detailed high-throughput analyses, such as relative expression patterns of the transcriptome and proteome, can be determined through high-throughput screening methods like RNA-sequencing (RNA-seq) and liquid chromatography-mass spectrometry (LC-MS). However, each of these methods is also destructive and only provides global, average measurements for the organoids. Determining spatial expression patterns, typically achieved through histochemistry and immunofluorescence, would be far more beneficial. The overall challenge with most current approaches is the lack of a true, live monitoring system, similar to measuring pH and temperature with a non-destructive probe, necessitating significant advancement in non-invasive, enhanced readouts. Although microfluidic devices are not ideal for scaling up the production of GBOs, the technology offers high-precision, low-volume manipulation for rapid, automated drug penetration and immunological interaction assays [188]. The implementation of microfluidic devices, perhaps in parallel, to run indirect multiomic assays during GBO development may overcome the enhanced readout gaps.

### 5.3. Logistics

Finally, the logistics behind establishing GBO biobanks and distributing GBOs globally are another often-overlooked challenge in the adoption of GBOs clinically and industrially. Long-distance transportation and biobanking relies on cryopreservation. However, cryopreservation of GBOs introduces a critical point of failure in the standardization pipeline. The core challenge lies in the variation of cryoprotectant (CPA) combinations, controlled cooling rates, and post-thaw recovery methods [132,136,192,193]. This lack of SOPs is detrimental because non-optimized protocols often result in selective cell death, where sensitive cell populations such as neuronal subtypes and GSCs are disproportionately damaged during the freeze/thaw cycle. If the cellular and structural composition of the organoid shifts due to poor or uneven cell recovery, the thawed model is no longer functionally identical to the one that was rigorously characterized, effectively invalidating all upstream standardization and QC efforts. The application of perfusion bioreactors is emerging as the long-term solution to enhancing the diffusion of nutrients to biological systems such as organoids. Perfusion bioreactors are emerging as the long-term solution to the oxygen diffusion limit to reduce necrotic core sizes as GBOs become larger. Most interestingly, they may also be strategically applied in the cryopreservation workflow [194]. Perfusion bioreactors have the unique ability to continuously deliver nutrients while simultaneously removing waste, ideal for CPA diffusion during freezing and essential nutrients during thawing. Lastly, cryopreservation may consider the incorporation of synthetic (i.e., polylactic acid or polyethylene glycol) hydrogel products for the purpose of retaining structural integrity. It is crucial that future cryopreserving protocols avoid using undefined hydrogel products both during freezing and after thawing to further avoid any issues in reproducibility.

## 6. Discussion: Current Tradeoffs and Needs in GBO Development

Review of the current literature revealed that the limitations in GBO translation of matured and functional structures (neurovascular unit and immune system) and cellular components (neurons) are a consequence of design choices prioritizing convenience and viability over true physiological relevance. This compromise can be broken into two interconnected oversights: pushing small micrometer-scale geometry of GBOs, and the reliance on undefined, animal-derived ECM hydrogels.

**Figure 2:**
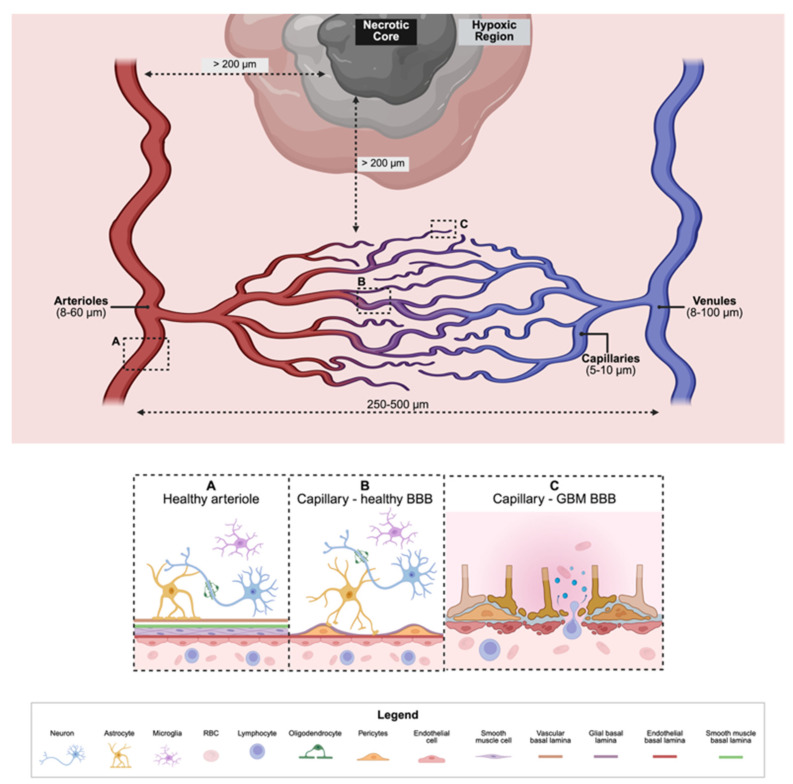
Glioblastoma (GBM) Neurovascular Unit (NVU). The distance between arterioles and venules is 250–500 μm, while the distance between capillaries is 40–50 μm. The diameter of arterioles, venules, and capillaries are 8–60 μm, 8–100 μm, and 5–10 μm, respectively. Capillaries form a vast network between arterioles and venules called the capillary bed. Healthy arterioles (**A**) and healthy capillaries (**B**) are well supported by endothelial cells (ECs), pericyte cells (PCs), and astrocyte cells (ACs) that communicate with neurons. Other glial cells such as oligodendrocytes and microglia support the BBB by myelinating neurons or clearing cell debris. The GBM blood–brain–barrier (BBB) is compromised once the basement membrane begins to degrade, reducing anchorage and causing anoikis for ECs, PCs, and ACs (**C**). These dying cells allow for the migration of unwanted cells and the secretion of neurotoxins, leading to inflammation. The distance from nutrient-delivering systems (i.e., arterioles and capillaries) to a necrotic core is ≥200 μm, beyond the oxygen diffusion limitations. This figure is not drawn to scale to highlight the overall features. Created in BioRender. Avera, A. (2025) https://BioRender.com/zrwozqf.

There is resistance in both academia and industry towards developing organoids larger than 400 μm diameter due to concerns over the limited viability, cost, and technical complexity of implementing enhanced readouts. The root cause of this size limitation is a 100–200 μm nutrient diffusion limit, beyond which a lack of oxygen risks inducing hypoxia or necrosis. While this size constraint is physiologically appropriate for modeling smaller tissue (e.g., a small intestinal crypt with a depth of 132–141 μm and diameter of 49–50 μm), it is not sufficient for recapitulating the native GBM TME [193,195,196,197]. The brain is unique, characterized by a generally hypoxic environment and a dynamic, low-oxygen profile in the GSC niche. Further emphasizing that modeling the GBM TME minimally requires the recapitulation of the NVU where GSCs are likely to reside and communicate between neurons, other glial cells, endothelial cells. The NVU geometry provides definitive scale constraints: capillary segments (5–10 μm diameter) are typically spaced 40–50 μm apart, while the larger arterioles and venules (8–100 μm diameter) are separated by 250 to 500 μm (Figure 2) [66,67,71,82,198,199,200]. Exhibition of necrosis is likely to occur beyond 200 μm from the closest nutrient transporting structure (i.e., capillaries, arterioles, and venules). The mechanisms by which hypoxia and necrosis drive GBM development, aggressiveness, and response to chemotherapies are well reviewed in the cited references [97,201,202]. Factoring in these physiological distances, the GBO must reach a minimum diameter of 1 mm, and likely closer to 2 mm, to establish a diffusion gradient severe enough to drive necrosis, not just initial hypoxia. Ultimately, the decision to avoid the necessary millimeter scale represents a sacrifice of pathological relevance for technical convenience.

The overreliance on undefined, animal-derived ECM such as Matrigel™ is an oversight many researchers are reluctant to change [160,203,204]. While stem cells are notoriously sensitive to their environment, the use of these matrices persists without a full understanding of their mechanism of action. Without a precise definition of components within animal-derived ECM hydrogels, the underlying biology remains obscured. More importantly, the use of exogenous matrices fundamentally inhibits the ability to investigate dynamic cell–ECM interactions and remodeling within the GBM TME. This interaction is not merely supportive; it is crucial to cell fate decisions, tumor invasion and progression, and the native attempt to restructure the NVU in response to nutrient deprivation [46]. Arguing that these biodegradable hydrogels are eventually replaced by the cell’s native ECM disregards the immediate influence of the external environment on a cell’s epigenetic state and mechanical memory. To achieve true physiological relevance, cells must be given the opportunity to generate and organize their own ECM to form native, structurally relevant TME components. Ultimately, a shift toward fully defined, chemically tunable hydrogels or avoiding hydrogel encapsulation altogether is necessary to enable the study of the inherent biology of cell–ECM signaling and to fully control the physical environment of the GBO.

The recurring theme of this review—the tradeoff between physiological relevance and technical convenience—has profound implications for translational efficiency. The absence of a necrotic core and associated steep hypoxic gradients means the organoid is likely to present an artificially sensitive profile during drug screening, where cells are not subject to the survival pressures of the in vivo TME. Furthermore, the use of undefined EMCs limits the ability to understand drug penetration and response, which are heavily influenced by matrix density and composition. The GBO standardization field must consistently review technical advancements and whether they align with the final goal of physiological relevance or technical convenience, and which one carries more weight for the problem at hand.

## 7. Conclusions

In summary, the advancement of in vitro modeling systems into glioblastoma organoids (GBOs) serves to bridge the gap between physiological relevance and accessibility not offered by conventional models (i.e., 2D monoculture or animal models). Despite much advancement, GBOs are still limited in functionally modeling key TME features, including neurovasculature, neural excitability, and immune response necessary for clinical translation. The constraints of GBO progression into functionality are less of a technical impossibility, but rather a lack of reproducibility driven by a preference for convenient standardized protocols. This trend has led to two fundamental oversights: the avoidance of millimeter-scale GBOs, and the persistence of undefined ECM hydrogels that obscure the study of critical tumor dynamics. The organoid field must not ignore these oversights and the root causes of them in future innovations. Once the development of GBOs becomes more reproducible across varying labs, the translation limitations (i.e., in functional vascularization, neural excitation, and immune system interactions) can be addressed in the emerging directions of co-culture systems.

## 8. Future Directions

The future of GBO research must focus on three interdependent engineering challenges. First, overcoming the scale limitation necessitates the development of advanced perfusion and bioreactor systems capable of reliably sustaining millimeter-scale constructs. Second, to unlock mechanistic studies, the field must transition entirely away from undefined components, including serums and ECM hydrogels. Lastly, these structural advancements must be coupled with enhanced, non-destructive, live readouts to fully characterize the complex cellular dynamics within the larger, opaque models. Each of these engineering challenges must be documented thoroughly to ensure standardization across all labs, allowing for global reproducibility of these models. Addressing these challenges will further enhance the translatability desperately needed in the GBO field.

## Figures and Tables

**Table 1:** Summary of Bioreactors for Glioblastoma Organoids.

Bioreactor Type	Bioreactor Geometry	Range of Shear (*τ*, Pa)	Uniformity of Shear	Working Volume Scalability (Scale Up or Out)	Refs.
Ultra-low attachment (ULA) plates	Static 2D surface (multi-well plates)	Negligible (~0 Pa)	N/A	Very low ≤2 mL (Scale out)	[176,177]
Stirred tank	Cylindrical	Low to moderate (<0.5 Pa)	Moderate. Highest shear is concentrated near the impeller or stirring element.	High, 50 mL to 1000–25,000 L (Scale up)	[117,139,178,179,180,181]
Rotating wall	Concentric	Very low and controlled (<0.01 Pa)	High. Homogenous shear distribution across the fluid volume due to constant rotation.	Medium, up to 500 mL (Scale out)	[180,182]
Perfusion	Hollow fiber/cartridge	Low to moderate (<0.1 Pa)	High. Designed for laminar flow.	Medium, up to 500 mL (Scale out)	[12,183]
Wave/shaking	Rectangular	Low to moderate pulsatile	Moderate to Low. Non-uniform; highest at the liquid-air interface due to wave direction.	Medium, 1 mL to 100 L (Scale out)	[182,184,185]

“Scale up” refers to increasing batch size with larger bioreactors. “Scale out” refers to maintaining small volumes with parallel flasks to increase production.

## Data Availability

No new data were created or analyzed in this study. Data sharing is not applicable to this article.

## Abbreviations

The following abbreviations are used in this manuscript:

2D	Two-Dimensional
3D	Three-Dimensional
ACs	Astrocyte Cells
ATRX	Alpha-thalassemia/intellectual disability X-linked
BBB	Blood-Brain-Barrier
CNS	Central Nervous System
CPA	Cryoprotective Agent
CSC	Cancer Stem Cell
CSPG	Chondroitin Sulfate Proteoglycan
ECM	Extracellular Matrix
ECs	Endothelial Cells
EGF	Epidermal Growth Factor
EGFRvIII	Epidermal Growth Factor Receptor vIII
ESCs	Embryonic Stem Cells
FBS	Fetal Bovine Serum
FDA	U.S. Food and Drug Administration
FGF	Fibroblast Growth Factor
GBM	Glioblastoma (multiforme)
GBO	Glioblastoma Organoid
GSC	GBM Stem Cell
HA	Hyaluronic Acid
IDH	Isocitrate Dehydrogenase
IDH1	Isocitrate Dehydrogenase 1
iPSCs	Induced Pluripotent Stem Cells
LC-MS	Liquid-Chromatography Mass-Spectrometry
LGG	Lower-Grade Glioma
NPCs	Neural Progenitor Cells
NSCs	Neural Stem Cells
NVU	Neurovascular Unit
OSKM	OCT3/4, SOX2, KLF4, and MYC
PCs	Pericyte Cells
PNNs	Perineuronal Nets
PSCs	Pluripotent Stem Cells
PTEN	Phosphatase and Tensin Homolog
QC	Quality Control
RGs	Radial Glial Cells
RNA-seq	Ribonucleic Acid Sequencing
SOM	Standardized Organoid Modeling (National Institute of Health Center)
SOP	Standard Operating Procedure
TAMs	Tumor Associated Microglia
TCPS	Tissue Culture Polystyrene
TERT	Telomerase Reverse Transcriptase
TME	Tumor Microenvironment
TNC	Tenascin-C
TP53	Tumor Protein P53
ULA	Ultra-low Attachment
